# Prognostic Value of SOX2 and NANOG Expression in Recurrent Oral Squamous Cell Carcinoma

**DOI:** 10.3390/cancers17071181

**Published:** 2025-03-31

**Authors:** Mohamed Falougy, Clara Taubitz, Mohab Ragab, Akshay Patil, Justus Jensen, Steffen Hoppe, Christiane Kümpers, Julika Ribbat-Idel, Dirk Rades, Samer George Hakim

**Affiliations:** 1 Department of Oral and Maxillofacial Surgery, Head and Neck Cancer Center, University Hospital Schleswig-Holstein, 23562 Lübeck, Germanysamer.hakim@uni-luebeck.de (S.G.H.); 2 Department of Internal Medicine II, Klinikum Rechts der Isar, TUM School of Medicine and Health, Technical University of Munich, 81675 Munich, Germany; 3 Derby Clinical Trials Support Unit, University Hospitals of Derby and Burton NHS Foundation Trust, Derby DE22 3NE, UK; 4 Institute of Pathology, University Hospital Schleswig-Holstein, 23562 Lübeck, Germany; 5 Department of Radiation Oncology, University Hospital Schleswig-Holstein, 23562 Lübeck, Germany; 6 Department of Oral and Maxillofacial Surgery, Helios Medical Center Schwerin, 19055 Schwerin, Germany

**Keywords:** oral squamous cell carcinoma, SOX2, NANOG, prognostic biomarkers, immunohistochemistry, overall survival, disease-free survival

## Abstract

Background: Recurrent oral squamous cell carcinoma (re-OSCC) poses a serious therapeutic challenge and is linked to poor survival outcomes. SOX2 and NANOG, key transcription factors in cancer stem cell biology, may drive tumor progression and therapy resistance. However, their prognostic value in re-OSCC and their relationship to adjuvant therapy remain unclear. Methods: We retrospectively analyzed a single-center cohort of 94 patients with re-OSCC treated with curative intent via (1) surgery alone, (2) surgery plus adjuvant radiotherapy (RT), or (3) surgery plus adjuvant radiochemotherapy (RCT). Tissue microarrays (TMAs) were constructed from matched primary and recurrent tumors and immunohistochemical (IHC) staining for SOX2, and NANOG was quantified using H-scores. Post-recurrence overall survival (prOS) and post-recurrence disease-free survival (prDFS) were evaluated using Kaplan–Meier analysis and Cox proportional hazards models. Results: SOX2 expression and survival: Elevated SOX2 expression (H-score > 14) in re-OSCC was significantly associated with improved prOS (*p* = 0.013) and prDFS (*p* = 0.026). Notably, patients who had received adjuvant therapy (particularly RCT) showed higher SOX2 levels in recurrent tumors compared to those treated with surgery alone. NANOG expression and therapy: NANOG expression declined markedly from primary to recurrent tumors (median H-score 42.2 vs. 8.7; *p* < 0.001). This decline was most pronounced in patients treated with surgery alone. Despite this dynamic change, NANOG expression did not correlate significantly with prOS or prDFS. Other prognostic factors include advanced tumor stage (rT2–rT4) and lymph node involvement (rN+/x)m which remained significant predictors of worse survival in the recurrent setting, regardless of adjuvant therapy. Conclusion: SOX2 overexpression in re-OSCC correlates with better survival, suggesting a unique prognostic role distinct from primary disease. Adjuvant therapy, especially RCT, appears to maintain or elevate SOX2 levels, potentially contributing to improved treatment response. In contrast, although NANOG expression decreases in recurrence, particularly in patients who undergo surgery alone, it does not significantly affect survival outcomes. These findings underscore the importance of context-specific biomarker assessments and provide a rationale for incorporating SOX2 status into personalized treatment strategies for re-OSCC.

## Simple Summary

Recurrent oral squamous cell carcinoma often leads to poor outcomes and limited treatment options. In this study, we examined two proteins, SOX2 and NANOG, which are involved in the growth and survival of cancer stem cells. We analyzed tumor samples from 94 patients to assess how the levels of these proteins change between the primary tumor and the recurrence, and whether these changes are linked to patient survival. We found that higher levels of SOX2 in recurrent tumors were associated with better survival. In contrast, NANOG levels decreased in recurrent tumors, especially in patients treated with surgery alone, but this decrease was not significantly associated with survival outcomes. These findings suggest that SOX2 could serve as a valuable marker for predicting prognosis in patients with recurrent oral squamous cell carcinoma. Identifying such markers may support more personalized treatment decisions and ultimately improve care for patients facing this aggressive disease.

## 1. Introduction

Oral squamous cell carcinoma (OSCC) is a prevalent and aggressive cancer type, which comprises about 90% of all malignancies in the oral cavity [1]. Despite the progress made in treatment strategies, OSCC continues to be associated with poor survival outcomes and high recurrence rates, underscoring the necessity of dependable prognostic biomarkers to enhance patient management [2,3].

In contrast to primary tumors, recurrent OSCC (re-OSCC) presents a distinctive set of challenges. The mechanisms that drive recurrence are not completely understood and the biology of recurrent tumors can differ significantly from that of the primary tumor [4]. The occurrence of lymph node metastases in the neck in oral cancer is associated with a worse prognosis, complicating the management of re-OSCC [5,6]. Higher tumor depth of invasion (DOI) is associated with a greater chance of lymph node metastasis, recurrence, and lower survival chances in early-stage oral squamous cell carcinoma [7]. Patients with re-OSCC frequently encounter limited therapy alternatives and reduced survival rates, necessitating an understanding of the expression patterns of these markers in recurrent illness [8]. As a result, the therapeutic strategies employed for re-OSCC are distinct from those employed for primary malignancies. Although surgical resection is the standard approach to initial OSCC management, recurrent cases frequently necessitate multimodal strategies, such as salvage surgery, re-irradiation, chemotherapy, or targeted therapies, that are tailored to the tumor’s changing biology and the patient’s previous treatments [9,10,11].

Re-OSCC frequently exhibits distinct biological characteristics compared to primary tumors, including increased resistance to apoptosis, enhanced invasiveness and alterations in gene expression, which contribute to its more aggressive nature [9,12,13]. Borsetto et al. examined key prognostic factors in the locoregional recurrence of OSCC, demonstrating that tumor size at initial diagnosis, the extent of recurrent disease, and positive surgical margins at primary surgery are strong predictors of poor overall survival [14]. Their results underscore the clinical challenge of re-OSCC, as patients with extensive locoregional recurrence have a substantially shorter survival rate than those with isolated local recurrence [15].

Cancer stem cells (CSCs) have emerged as key participants in tumor initiation, development, metastasis, and recurrence. These cells have self-renewal capacities and the ability to differentiate into various tumor types, making them important targets for therapeutic intervention [16]. Abnormal expression of transcription factors SOX2 (SRY-related HMG-box gene 2), NANOG, and OCT4 is known to have different functions in tumor initiation and metastasis [17,18,19,20]. NANOG is critical for maintaining CSC self-renewal, regulating cell proliferation via cyclin D interactions, decreasing E-cadherin to increase migration and disrupting p53 to avoid apoptosis [21]. SOX2, located on 3q26.3–q27, is identified as a major oncogene in OSCC [13], associated with tumor proliferation, metastasis, therapeutic resistance, and the facilitation of epithelial–mesenchymal transition (EMT) [17,22,23].

High expression of these markers is frequently related to higher tumor aggressiveness and poor clinical outcomes [12,24]. The significance of SOX2 in radioresistance is intricate, with research yielding conflicting results. Some study indicates that SOX2 amplifies the effects of irradiation, resulting in a better prognosis for patients receiving radiotherapy, whereas other evidence suggests that suppressing SOX2 may generate a radioresistant phenotype [25]. Nanog signaling augments radioresistance in ALDH-positive breast cancer cells by increasing ALDH activity and promoting double-strand break (DSB) repair, possibly through the Notch1 and Akt pathways [26]. While the involvement of SOX2 and NANOG in primary OSCC has been explored [4,11,13,27,28,29,30], their prognostic significance in re-OSCC remains largely understudied.

The purpose of this study is to examine the expression patterns of SOX2 and NANOG in both primary and re-OSCC, with an emphasis on how adjuvant therapy affects their expression dynamics. Furthermore, we evaluate the prognostic importance of these markers by examining their expression in re-OSCC and their relationship with post-recurrence overall survival (prOS) and post-recurrence disease-free survival (prDFS) in a well-defined, prospectively collected single-center cohort. This study seeks to improve risk assessment and facilitate the development of customized treatment regimens for patients with re-OSCC by clarifying the roles of SOX2 and NANOG in OSCC progression and medication response.

## 2. Methods

### 2.1. Patient Cohort

**Figure 1:**
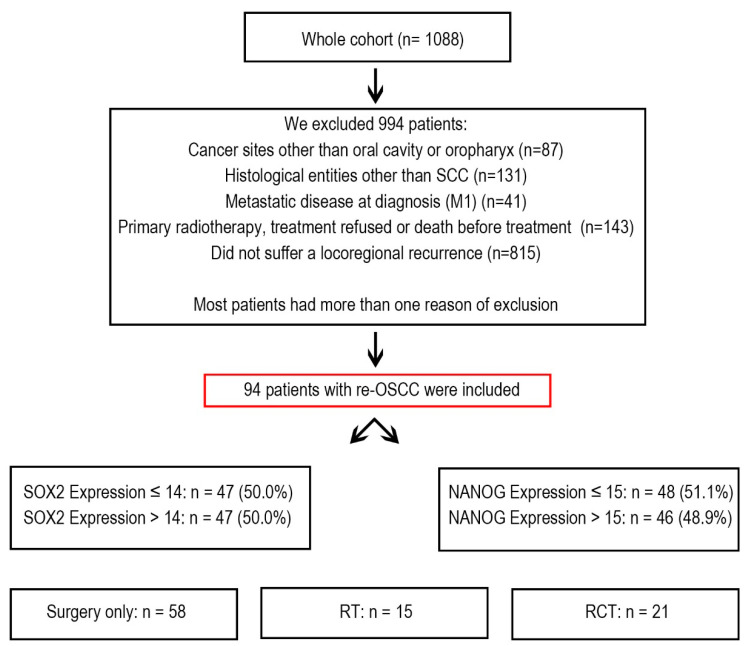
Flowchart illustrating patient selection and stratification. Cancer patients treated at the University Hospital of Lübeck were screened, with the majority excluded based on predefined criteria. The final cohort consisted of patients with re-OSCC who underwent curative treatment. These patients were further categorized based on SOX2 and NANOG expression levels and stratified by treatment modality, including surgery alone, RT, and RCT.

Between 1992 and 2019, the Department of Maxillofacial Surgery at the University Medical Centre of Lübeck in Germany treated 1088 cancer patients. From this group, we selected 94 patients who developed re-OSCC and underwent treatment with curative intent. These patients underwent multiple treatments, including surgery, radiotherapy (RT), chemoradiotherapy (RCT), or a combination of these (Figure 1).

Patients were excluded if they had oropharyngeal carcinoma, a histological diagnosis other than squamous cell carcinoma, metastatic disease at the time of diagnosis, or if they declined treatment or passed away before therapy could begin. Additionally, patients who did not develop locoregional recurrence were excluded from the study.

All eligible patients were registered in a structured recall system to ensure continuous follow-up for five years after treatment. Follow-up appointments were scheduled every three months for the first two years, then every six months following. At each visit, demographic information, risk factors, clinical tumor features, and therapy details were carefully documented. Each patient’s general health state was assessed using the Charlson Comorbidity Index (CCI) [31], incorporating tumor staging and additional prognostic variables into the cohort analysis [32].

#### Tissue Microarray and Immunohistochemical Staining

Before analysis, each tissue punch was visually inspected for tissue folds, staining artifacts, low tissue quality, or insufficient tumor content. Any punches that did not meet quality standards were excluded.

Tissue microarrays (TMAs) were constructed using a manual Tissue Arrayer MTA-1 (AlphaMetrix Biotech, Rödermark, Germany). Tumor regions from formalin-fixed, paraffin-embedded (FFPE) tissue blocks were cut out using 1.2 mm punches and subsequently transferred to recipient blocks. To guarantee the precise representation of intratumoral intricacy, three punches from each tissue block were incorporated into the TMAs. The recipient blocks were sliced to a thickness of 4 μm using a Microm HM355S microtome (Thermo Scientific, Boston, MA, USA). The slices were subsequently positioned on slides, immersed in a 50 °C water bath and incubated in a 60 °C hot air oven for 1 h prior to additional processing. Immunohistochemical (IHC) staining protocols were modified from prior studies and refined with positive and negative controls to guarantee dependable outcomes [32]. Antibodies used in the study are listed in Table 1.

**Figure 2:**
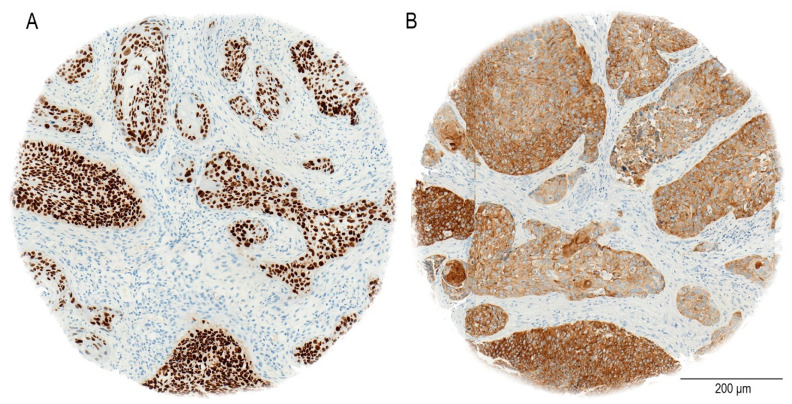
An example of an immunohistochemical staining of OSCC TMA cores (**A**) SOX2 expression with nuclear staining. (**B**) NANOG expression with cytoplasmic staining. Bar = 200 µm.

The staining procedure was entirely automated utilizing the Ventana BenchMark system (Roche, Basel, Switzerland). In our study, the immunohistochemical analysis demonstrates that SOX2 exhibits nuclear staining, as previously reported [33], NANOG shows a cytoplasmic staining pattern in oral OSCC patients, as seen in our cohort (Figure 2). In particular, NANOG has strong cytoplasmic staining in a considerable majority of OSCC cases, in contrast to the nuclear localization seen in germ cell malignancies [34,35].

Antigen retrieval for SOX2 staining was conducted using heat-mediated retrieval in sodium citrate buffer (pH 6.0) at 92 °C for 32 min. The sections were treated with anti-SOX2 antibody (dilution 1 µg/mL) at ambient temperature, and thereafter, detected with a biotin-conjugated secondary antibody and streptavidin–peroxidase solution. Counterstaining was conducted with hematoxylin.

Antigen retrieval for NANOG staining was performed using sodium citrate buffer (pH 6.0) at 92 °C for 32 min. The slides were treated with anti-NANOG antibody at a dilution of 1:1000 to 1:2500 at room temperature. After the primary antibody incubation, a biotin-conjugated secondary antibody was administered for 30 min, succeeded by streptavidin–peroxidase incubation. The staining was observed utilizing 3′,3-diaminobenzidine tetrahydrochloride (DAB) and counterstained with hematoxylin.

Positive controls for SOX2 included human glioblastoma tissue and NCCIT (human pluripotent embryonal carcinoma) cell line samples. Positive controls for NANOG staining included NCCIT cells and embryonic carcinoma tissue.

Negative controls were prepared by omitting the primary antibodies during the staining process to confirm specificity.

For digital analysis, slides were scanned using a Ventana iScan HT Scanner (Ventana, Tucson, AZ, USA). Image analysis was conducted using the open-source software QuPath (Version 0.2.3) [36]. TMA cores were selected using the integrated TMA dearrayer function in QuPath software. Patient data were imported from prepared Excel spreadsheets to ensure that each core was correctly matched to the corresponding patient. Regions of interest, including tumor cells, immune cells, and stromal compartments, were manually annotated for analysis. Positive cell detection was performed using the Optical Density Sum (OD Sum) method to reduce nucleus fragmentation. Parameters such as requested pixel size, background radius, median filter radius, nucleus size thresholds, intensity threshold, and cell expansion were optimized to ensure accurate detection of cells. The smoothed measurement heat maps function was subsequently generated to visualize classification-relevant cellular features. A Random Forest classifier was trained based on the annotated regions to facilitate accurate automated classification of tumor cells. Finally, cellular measurements were exported via the TMA Data Viewer, allowing for the calculation and validation of the H-score.

We utilized QuPath for image processing and H-score quantification, leveraging its demonstrated reliability in several tumor biology studies [22,32,37]. The cited research validates the reproducibility of QuPath for H-score assessments across different tumor contexts. Two independent pathologists supervised the process to minimize subjectivity.

Digitized image files were imported into QuPath as .tif files for further examination on a Windows 10-based system with a 12.3″ display (2736 × 1824 pixels resolution).

### 2.2. Statistical Analysis

Descriptive statistics were summarized using frequencies, percentages, medians, and ranges. Continuous data are presented as means (SD) and medians with an interquartile range (IQR) for a comprehensive representation of data distribution.

For survival analysis, the Kaplan–Meier method was applied to estimate prDFS and prOS probabilities.

prDFS was defined as the time from the first recurrence until a second recurrence, death, or last follow-up, whichever occurred first. During this period, the patient remained disease-free.

prOS was defined as the time from the first recurrence to death from any cause or the last follow-up date, whichever came first.

Group comparisons were conducted using univariate analysis with the log-rank test and Schoenfeld residuals were examined to verify the proportional hazards assumption.

To determine optimal cutoff points for marker expression, the “rolr” package was used, specifically its “rhier” function, which identifies the best two-group split based on survival outcomes. Cutoff points were selected based on median values where appropriate.

Kaplan–Meier plots were generated using a combination of “survival”, “survminer”, “ggplot2”, and “plotly” packages. Hazard ratios (HR), confidence intervals (CI), and *p*-values were estimated using the “coxph” function from the “survival” package.

All statistical tests were two-tailed, with significance set at *p* < 0.05. Results were reported with 95% confidence intervals (CI). Statistical analyses were conducted using R Core Team (2023), R: A Language and Environment for Statistical Computing (R Foundation for Statistical Computing, Vienna, Austria) (https://www.R-project.org/ (accessed on 29 July 2024)).

This study is among the first to examine survival outcomes in recurrent OSCC by analyzing SOX2 and NANOG expression using semi-automated immunohistochemical marker analysis with QuPath software. The applied method assessed post-recurrence survival in high-risk groups by correlating standardized H-score-based IHC expression with clinical follow-up data.

**Figure 3:**
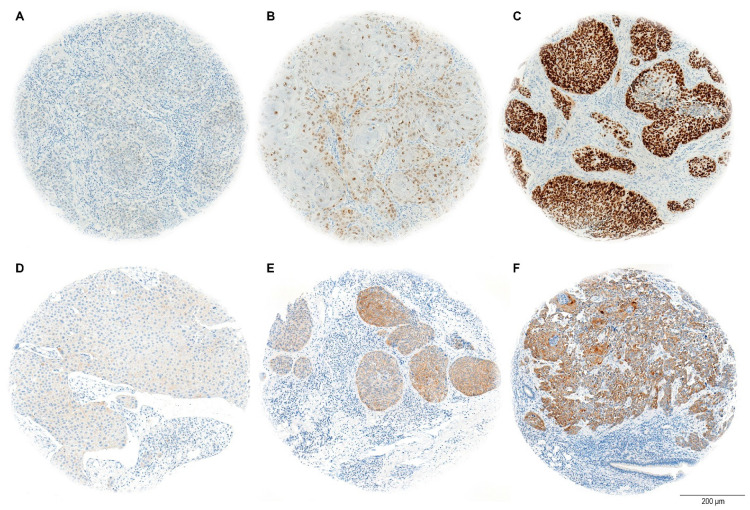
Representative images of stained TMA cores for SOX2 (**A**–**C**) and NANOG (**D**–**F**), showing weak (**A**,**D**), moderate (**B**,**E**), and strong (**C**,**F**) immunohistochemical staining. The scale bar indicates 200 µm.

The H-score evaluation of immunostaining quantifies protein expression in tissue samples, with a maximum possible score of 300. This score is calculated by adding the weighted contributions of stained cells based on their intensity: strongly stained cells are weighted at 3, moderately stained cells at 2, and weakly stained cells at 1. Cells without expression are weighted at 0 (Figure 3). The percentage of positively stained tumor cells was considered relative to the total tumor area [38].

Additionally, a direct comparison of antibody expression between primary and recurrent tumors was performed, categorized according to the specific therapeutic approaches (surgery alone, adjuvant radiotherapy, or radiochemotherapy), using the nonparametric Wilcoxon signed-rank test, as the data were not normally distributed.

#### Ethics

Upon admission, all participants signed consent forms authorizing the collection and anonymous use of their data for academic research. The study (ID: 16-272A) was approved by the ethics review committee of the University of Lübeck in April 2017.

## 3. Results

### 3.1. The Floor of the Mouth Is the Most Common Site of Recurrence Among OSCC Patients

This study examined a cohort of 94 patients with re-OSCC, describing demographic and clinical characteristics that highlight issues of recurring disease (Table 2). The median patient age was 62 years, with the majority (68%) being male. Notably, 78% were previous or current smokers, with 60% reporting heavy alcohol usage, indicating high exposure to known carcinogens. Comorbidities were distributed as follows: 63% had a Charlson Comorbidity Index (CCI) of 0, whereas 37% had a CCI of 1. The most common recurrence site was the floor of the mouth (41%) and a majority (68%) showed lymph node involvement at recurrence (rN+/x). At the tumor level, 31% of patients had rT4 lesions and 11% had rT3, emphasizing the aggressive nature of re-OSCC; only 8.8% presented with distant metastases (rM1). These variables collectively indicate a population with advanced, high-risk recurring disease, emphasizing the intricate therapeutic decision-making required for this group.

### 3.2. High SOX2 Expression Predicts Better Survival, While NANOG Shows No Impact

Survival data revealed that 71% of patients had died from any cause (Table 3). Mortality was higher among patients with a SOX2 H-score of ≤14 (83%) compared to those with scores >14 (60%). Deaths attributed specifically to oral cancer were observed in 70% of the low SOX2 group, whereas this was lower at 51% in the high SOX2 group. In relation to prDFS, 43% of the patients showing low SOX2 expression died from oral cancer, in contrast to 34% in the high SOX2 cohort. Locoregional recurrence rates were similar between groups, occurring in 33% of patients.

Analysis of NANOG expression revealed that individuals with an H-score ≤ 15 had a greater overall mortality rate (77%) compared to those with scores > 15 (65%) (Table 4). Mortality from oral cancer was more prevalent in the low NANOG group (63%) than in the high NANOG group (59%). The percentage of patients who died to oral cancer during prDFS was comparable between the NANOG expression groups. Locoregional recurrence was observed in 29% of patients exhibiting low NANOG expression and 37% of those with elevated expression.

### 3.3. SOX2 and NANOG Show Divergent Prognostic Roles

Cox regression analysis (Table 5 and Table 6) identified larger recurrent tumor size (rT2–rT4) and lymph node involvement (rN+/x) as significant predictors of worse outcomes in both prDFS and prOS. In Table 5, which focuses on SOX2 expression, patients with rT2 had a hazard ratio (HR) of 3.51 (95% confidence interval [CI]: 1.53–8.07) for prDFS and 3.56 (95% CI: 1.36–9.31) for prOS, while rT3 (HR: 3.01 for prDFS; HR: 5.70 for prOS) and rT4 (HR: 2.48 for prDFS; HR: 3.58 for prOS) further demonstrated a significantly increased risk. Similarly, Table 6, which examines NANOG expression, confirmed these associations, with rT2 showing an even higher HR of 4.77 (95% CI: 2.07–11.0) for prDFS and 4.91 (95% CI: 1.86–13.0) for prOS. The trend persisted for rT3 (HR: 3.19 for prDFS; HR: 5.93 for prOS) and rT4 (HR: 3.08 for prDFS; HR: 4.39 for prOS), reinforcing that advanced tumor stage at recurrence is strongly linked to poorer survival outcomes in OSCC. Furthermore, lymph node involvement (rN+/x) was consistently associated with increased mortality risk, with HR values of 1.95 for prDFS (*p* = 0.021) and 2.87 for prOS (*p* = 0.001) in Table 5 and similar effects observed in Table 7 (HR: 1.92 for prDFS; HR: 2.85 for prOS).

Regarding biomarker expression, SOX2 and NANOG displayed contrasting prognostic significance. In Table 5, SOX2 overexpression (H-score > 14) was associated with a significantly lower risk of death (HR: 0.54, 95% CI: 0.31–0.94, *p* = 0.030), suggesting its potential as a favorable prognostic biomarker in recurrent OSCC. In contrast, Table 7 demonstrated that NANOG expression (H-score > 15) was not significantly correlated with either prDFS (HR: 1.11, *p* = 0.7) or prOS (HR: 1.06, *p* = 0.8), indicating its limited prognostic value. These findings highlight that while larger tumor size and lymph node involvement remain the strongest predictors of poor prognosis, SOX2 overexpression may confer a survival advantage, whereas NANOG expression does not appear to influence recurrence-related outcomes.

### 3.4. SOX2 Stays Stable, NANOG Expression Decreases in Primary vs. Recurrent Tumors Across Treatments

**Figure 4:**
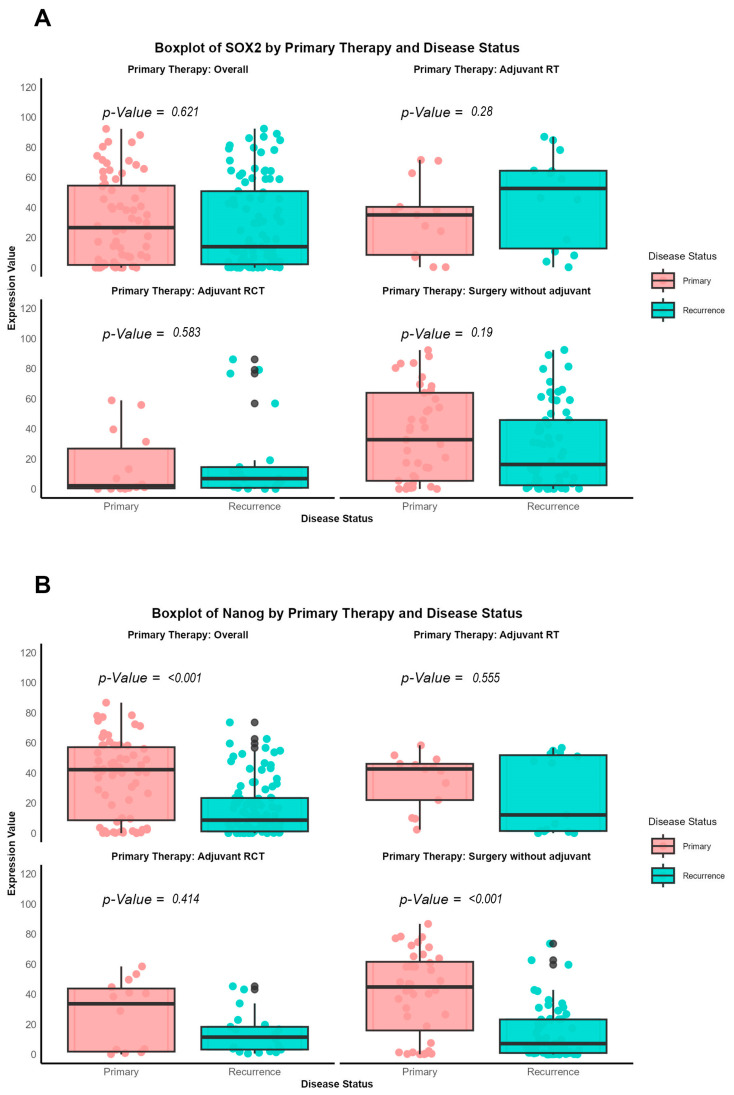
Box plots comparing SOX2 (**A**) and NANOG (**B**) expression between primary and recurrent OSCC tumors, stratified by therapy. Statistical differences were detected by the Wilcoxon rank sum test.

A comparison of SOX2 and NANOG expression between primary and recurrent tumors across different treatment modalities (Table 7 and Table 8 and Figure 4) showed that SOX2 expression did not significantly differ between primary (median expression: 26.631, IQR: 1.832–54.559) and recurrence tumors (median expression, IQR: 2.270–50.861; *p* = 0.621). However, in patients who received adjuvant radiotherapy (RT), SOX2 expression was higher in recurrent tumors (median expression, IQR: 12.753–64.455) compared to primary tumors (median expression: 35.061, IQR: 8.521–40.421; *p* = 0.280). In contrast, NANOG expression significantly decreased from primary to recurrent tumors in the overall cohort (primary median expression: 42.221, IQR: 8.585–57.148; recurrent median expression: 8.652, IQR: 1.177–23.368; *p* < 0.001). This decrease in expression was most notable in patients who underwent surgery without adjuvant therapy (primary median expression: 44.814, IQR: 15.930–61.558; recurrent median expression: 7.187, IQR: 0.943–23.241; *p* < 0.001). These findings suggest that treatment modalities may influence the expression patterns of SOX2 and NANOG in recurrent OSCC.

We investigated the effects of RT and RCT on tumor biology, specifically CSC features. Patients who underwent surgery without adjuvant therapy served as the control group because their tumors had not been exposed to RT or RCT. This method enables the assessment of tumor development without the altering effects of adjuvant therapy. Importantly, all primary tumor samples were acquired from patients who had not previously undergone adjuvant therapy, resulting in a consistent baseline for comparing primary and recurrent disease.

### 3.5. SOX2 Overexpression Significantly Improves prOS and prDFS

**Figure 5:**
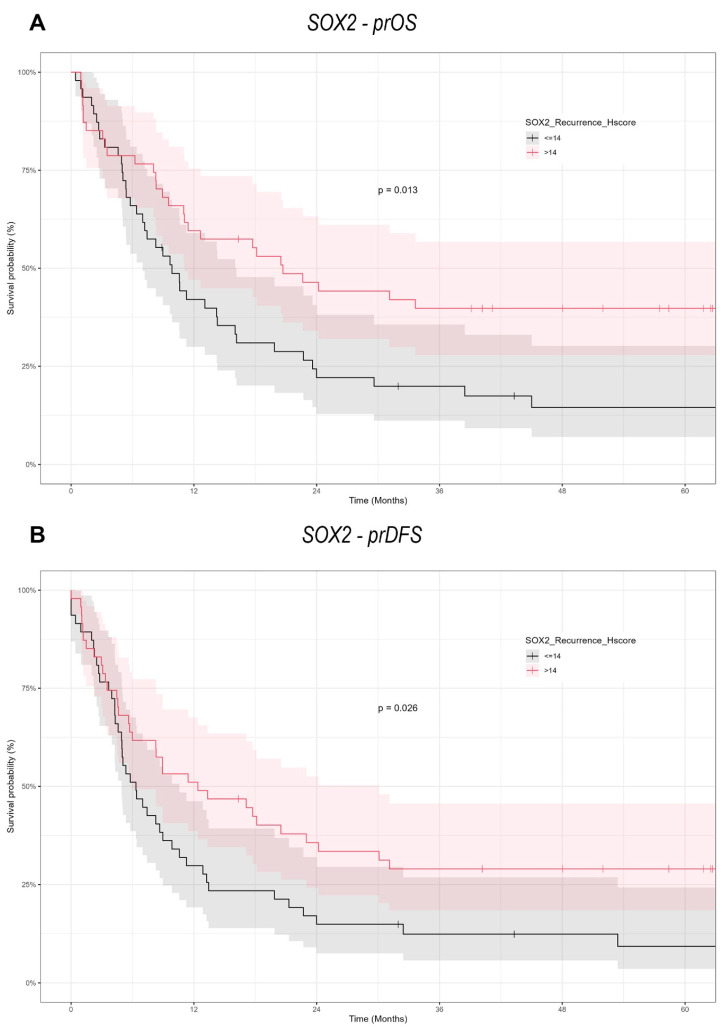
Kaplan–Meier survival curves illustrating the impact of SOX2 expression on survival outcomes in re-OSCC patients. (**A**) Curves for prOS showing significantly worse survival in patients with lower SOX2 expression (H-score ≤ 14). (**B**) Curves for prDFS similarly indicating poorer survival associated with lower SOX2 expression levels.

**Figure 6:**
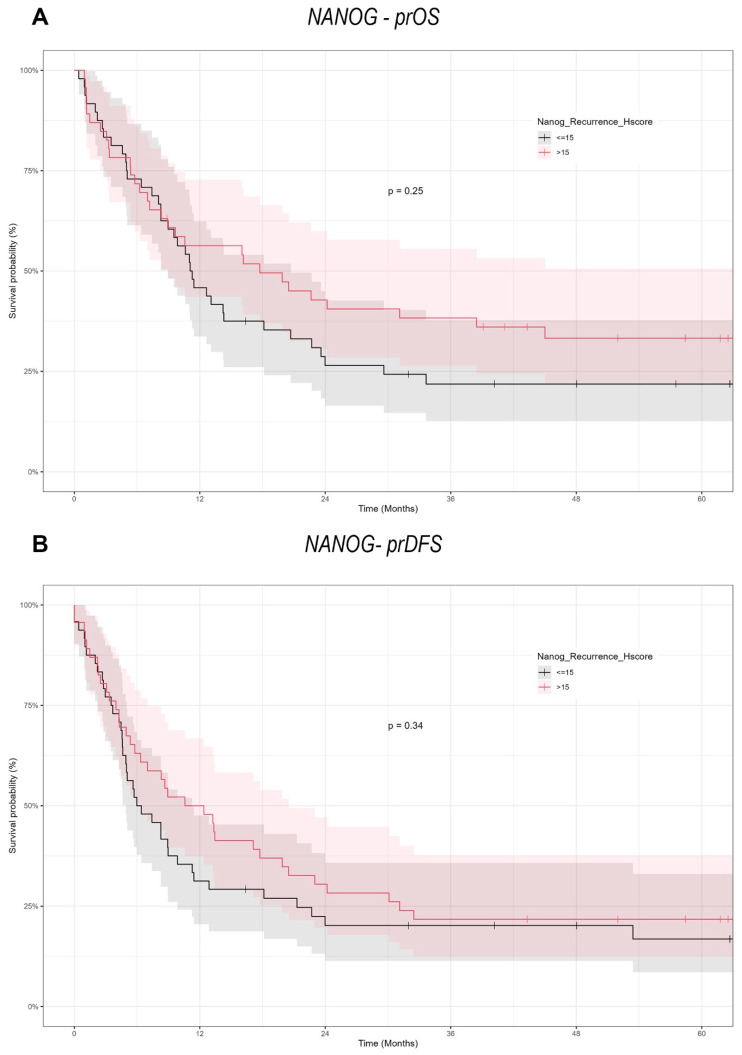
Kaplan–Meier survival curves illustrating the impact of NANOG expression on survival outcomes in re-OSCC patients. (**A**) Curves for prOS showing no significant difference in survival based on NANOG expression levels (H-score ≤ 15 vs. >15). (**B**) Curves for prDFS also demonstrating no significant survival differences related to NANOG expression.

The Kaplan–Meier survival analysis (Figure 5 and Figure 6) underscored the predictive significance of SOX2 and NANOG expression. Patients exhibiting elevated SOX2 expression (H-score > 14) had markedly prolonged progression-free overall survival compared to those with decreased expression (H-score ≤ 14), with a log-rank test *p*-value of 0.013. In a similar manner, elevated SOX2 expression was associated with prolonged prDFS (*p* = 0.026). NANOG expression did not significantly influence prOS (*p* = 0.25) or prDFS, as the survival curves for high (>15) and low (≤15) NANOG expression groups were almost indistinguishable.

SOX2 expression in recurrent cancers is associated with better survival, where a SOX2 H-score above 14 is connected to reduced mortality and prolonged progression-free overall survival (prOS). Increased tumor size and regional lymph node involvement forecast unfavorable outcomes. NANOG expression exhibited no significant effect on survival but reduced markedly in recurring tumors, especially following surgery alone. These data indicate SOX2 as a possible predictive biomarker, but alterations in NANOG expression may signify tumor biology in re-OSCC.

## 4. Discussion

A growing body of evidence highlights the complexity of re-OSCC, which frequently exhibits more aggressive biological behavior and poorer survival outcomes than primary tumors. The underlying mechanisms causing these differences are only partially understood. In this context, understanding the prognostic roles of pluripotency transcription factors, particularly SOX2 and NANOG, in re-OSCC is critical, given their established links to CSC biology and tumor progression.

Our findings provide a notable contrast to the prevailing literature, which often associates high expression of pluripotency-associated transcription factors (NANOG and SOX2) with increased metastatic potential in tumor cells. In the context of re-OSCC, however, we observed that elevated SOX2 expression correlates with better prognosis, including improved prOS and prDFS. This discrepancy may stem from the distinct biological features of recurrent versus primary malignancies, suggesting that markers like SOX2 could behave differently depending on a tumor’s evolutionary history.

The involvement of SOX2 and NANOG in OSCC has been thoroughly examined in primary tumors. Our study is focused on recurrent oral cavity tumors. This distinction is crucial, as prior research frequently grouped HNSCC from different anatomical locations (e.g., oropharynx, larynx), which could suggest varying biological behaviors [25,39,40,41]. By refining our cohort to re-OSCC, we want to find out whether the expression patterns of these CSC markers vary at recurrence and how this may influence patient outcomes.

This study is especially important for CSC-marker research because it suggests that recurrence-specific tumor biology influences the prognostic implications of NANOG and SOX2. Although these factors are generally implicated in stemness and tumor progression, their roles may differ across clinical stages and disease contexts [27].

Several investigations on SOX2 in OSCC have yielded mixed or conflicting prognostic results. Züllig et al. examined 120 patients with early-stage OSCC, investigated SOX2 by immunohistochemistry on TMA, assessed by an Intensity/Reactivity Score (IRS ≥ 8). Elevated SOX2 expression was associated with an absence of lymph node metastasis, indicating a potential protective function in original tumors. The present investigation on re-OSCC, utilizing the H-score for SOX2 assessment, indicates that overexpression correlates with enhanced post-recurrence survival, underscoring its prognostic relevance in primary versus recurrent disease and accentuating the necessity for context-specific biomarker evaluation [13].

Freier et al. examined SOX2 changes in OSCC, revealing gene copy number amplification in 52% of 223 tumors and elevated SOX2 protein expression in 18% of 271 cases via immunohistochemistry. Their findings indicate that SOX2 functions as a proto-oncogene in primary OSCC [42].

Sacco et al. examined 24 OSCC patients, assessing SOX2 expression using qRT-PCR and Western blot analysis. SOX2 was found to be elevated in both the tumor core and close margin, correlating with tumor size and lymph node involvement, indicating a potential role in local dissemination [43]. However, our data reveals a distinct prognostic significance in re-OSCC, wherein SOX2 overexpression is associated with enhanced post-recurrence survival, indicating a change in its biological role between primary and recurrent disease.

Steen et al. examined 222 OSCC patients, including 75 matched lymph node metastases and 33 recurrent tumors, to measure SOX2 expression using immunohistochemistry on tissue microarrays and entire histological slices. The authors used QuPath for digital pathology scoring, with an H-score cutoff of 12 (low: 0–11, high: ≥12). Their findings revealed no significant relationship between SOX2 expression and overall survival, whereas SOX9 overexpression and the SOX2^low^SOX9^high^ subgroup were associated with a poor prognosis. Our study, using an H-score cutoff of 14 (low: ≤14, high: >14), demonstrated that SOX2 overexpression in re-OSCC was linked with improved survival. These disparities demonstrate how variations in cutoff criteria and tumor context may influence SOX2’s prognostic relevance, stressing the importance of standardizing scoring techniques in OSCC research [22].

In other malignancies, such as colorectal cancer (CRC) and small cell lung cancer (SCLC), SOX2 often correlates with worse survival, chiefly through its roles in promoting stemness, chemoresistance, and EMT [44,45]. These differences further highlight the context-dependent roles of SOX2 and the necessity of examining its effects in specific clinical contexts, such as re-OSCC.

NANOG, another important stemness factor, is similarly involved in CSC maintenance and therapeutic resistance [27,30,46]. However, its prognostic importance in OSCC continues to be a topic of debate. Some studies correlate NANOG overexpression with OSCC progression, lymph node metastases and cisplatin resistance [30,47]. Others claim more complex roles, such as potential tumor-suppressive effects or involvement primarily in the early phases [27]. Our results did not reveal a significant association between NANOG expression and survival in recurrent OSCC, suggesting its impact may be more relevant in tumor initiation or primary disease settings than at recurrence. For instance, while one group found that NANOG and Midkine overexpression correlated with advanced stages and poor prognosis, we did not observe a similar relationship in re-OSCC [48]. Similarly, Kashyap et al. identified NANOG as a driver of OSCC aggressiveness and cisplatin resistance, yet our data imply that in the recurrent context, NANOG expression does not predict survival [47]. In addition, studies by Lee et al. highlight that the co-expression of NANOG and mutant p53 is particularly detrimental for survival in primary OSCC [28]. However, such combined molecular interactions were not explored in our study of re-OSCC, pointing to additional complexities in NANOG-mediated pathways that might require further investigation.

While most evidence portrays SOX2 as an oncogenic driver in primary OSCC, our study suggests it may serve as a favorable prognostic biomarker in re-OSCC. One hypothesis is that elevated SOX2 expression at recurrence could reflect a more differentiated, therapy-responsive subpopulation, rather than one prone to unchecked aggression. Alternatively, SOX2 might activate pathways that specifically enhance sensitivity to second-line treatments or radiotherapy at recurrence.

In support of this, several meta-analyses have shown that SOX2 expression in non–small cell lung cancer (NSCLC) and esophageal squamous cell carcinoma (ESCC) correlates with better survival outcomes [49,50,51]. A similar pattern may be emerging in re-OSCC. By contrast, NANOG seems more strongly implicated in primary tumor aggressiveness and therapy resistance, particularly in facilitating radioresistance and cisplatin resistance [26,52].

Our findings further indicate that SOX2 expression is higher in recurrent tumors of patients who received adjuvant radiotherapy, implying a potential link between SOX2 status and therapy responsiveness. Silencing SOX2 or suppressing related signals, such as β-catenin and hedgehog, may improve therapeutic efficacy and overcome resistance in re-OSCC [41,53,54,55].

SOX2 is well known for its relationship with a number of druggable pathways, the most notable being the Notch pathway. According to Hao Wu et al., Notch1 and SOX2 have a positive feedback regulation mechanism, showing a strong relationship between the two components. This link suggests that targeting the Notch pathway could be a possible therapeutic option in SOX2-related diseases like glioma [56]. NANOG interacts with a variety of druggable pathways, including HA-CD44, STAT3, PKC, and metabolic pathways, which all contribute to drug resistance and tumor progression. Focusing on these interactions may enable novel treatment techniques to improve chemosensitivity and more effectively treat cancer [57,58,59].

Despite the fact that this study offers valuable insights into the prognostic significance of SOX2 and NANOG in re-OSCC, it is important to recognize its drawbacks. Initially, the data were obtained from a single-center cohort, which could affect the overall validity of the results. In order to verify these findings, it is necessary to conduct multicenter studies with more diverse and extensive patient populations. Secondly, the study lacks to provide mechanistic insights into how SOX2 influences tumor biology or treatment response, despite the fact that it demonstrates an association between SOX2 expression and improved survival. In order to investigate the functional role of SOX2 in re-OSCC, future research should incorporate in vitro and in vivo models. Furthermore, the investigation mainly utilizes immunohistochemical analysis, neglecting molecular profiling methodologies such as gene expression analysis or RNA sequencing, which could offer a more thorough comprehension of the regulatory pathways that regulate SOX2 and NANOG expression.

A data-driven methodology employing survival-based optimization established the H-score threshold for SOX2 in our samples. This threshold successfully categorized patients into prognostically separate categories, although it may not be universally relevant to other cohorts. Distinct datasets may demonstrate divergent distributions of SOX2 expression, requiring validation in different cohorts to ascertain its prognostic relevance. The fact that the majority of re-OSCC tumors demonstrate diminished SOX2 expression (median H-score ~14) underscores the significance of context-specific cutoff determination in biomarker research. The same applies to NANOG. At present, there is no universal agreement on the ideal H-score cutoffs for both SOX2 and NANOG in re-OSCC. The survival-based cutoff selection guarantees statistical validity, although it may not accurately represent biological thresholds that have practical relevance. Future research should focus on standardizing cutoff definitions utilizing multi-institutional data.

In conclusion, our study demonstrates the context-dependent functions of SOX2 and NANOG in re-OSCC. In contrast to many primary OSCC reports, which tend to indicate a poor prognosis due to SOX2 overexpression, we observed a significant positive correlation between SOX2 expression and prOS/prDFS in re-OSCC cells. On the other hand, NANOG lacked prognostic significance in this recurrent setting, which means that it may be more critical during the early stages of tumorigenesis. These results reinforce the necessity of conducting larger, multicenter trials to independently confirm the recurrent disease-specific functions of these CSC markers and to determine whether targeted inhibition or modulation of SOX2 and NANOG can enhance outcomes in re-OSCC. It will be essential to learn about the process in which CSC-related factors transition between primary and recurrent disease in order to create more personalized, effective therapeutic strategies for OSCC.

## Figures and Tables

**Table 1:** Markers used for the immunohistochemical staining of the tissue blocks.

Antibody	Isotype	Staining Pattern	Company	Concentration/ Dilution
SOX2	IgG (Rabbit polyclonal)	nuclear	Abcam	1 µg/mL
NANOG	IgG (Rabbit monoclonal)	cytoplasmic	Abcam	1:1000–1:2500

**Table 2:** Baseline demographics of all patients and their tumor characteristics.

Characteristic	N = 94
Median Age	62 (52, 71)
Median Age at recurrence	63 (53, 72)
Sex	
Female	30 (32%)
Male	64 (68%)
CCI score	
0	59 (63%)
1≤	34 (37%)
Missing	1
Smoking status	
Never	20 (22%)
Former or current	69 (78%)
Missing	5
Alcohol consumption	
None or moderate	35 (40%)
Excessive	52 (60%)
Missing	7
Site of recurrence	
Anterior tongue	11 (12%)
Cheek/vestibule/retromolar	13 (14%)
Floor of mouth	39 (41%)
Lip	2 (2.1%)
Neck only	15 (16%)
Oropharynx	11 (12%)
Palate	3 (3.2%)
rT	
rT1	24 (26%)
rT2	16 (18%)
rT3	10 (11%)
rT4	28 (31%)
rTx	13 (14%)
Missing	3
rN	
rN0	29 (32%)
rN+/x	63 (68%)
Missing	2
rM	
rM0/x	83 (91%)
rM1	8 (8.8%)
Missing	3
Resection margins	
R0	19 (68%)
R1/2/x	9 (32%)
Missing	66
Grade	
Well	6 (8.8%)
Moderate	40 (59%)
Poor	22 (32%)
Missing	26

**Table 3:** Survival events in the patients’ cohort according to the H-score-based cutoff by SOX2 expression.

Characteristic	Overall, N = 94 ^1^	≤14, N = 47 ^1^	>14, N = 47 ^1^
Death of any cause			
Alive or censored	27 (29%)	8 (17%)	19 (40%)
Dead	67 (71%)	39 (83%)	28 (60%)
Cause of death			
Alive or censored	27 (29%)	8 (17%)	19 (40%)
Death from oral cancer	57 (61%)	33 (70%)	24 (51%)
Death from other causes	10 (11%)	6 (13%)	4 (8.5%)
prDFS			
Censored	18 (19%)	5 (11%)	13 (28%)
Death from oral cancer	36 (38%)	20 (43%)	16 (34%)
Death from other causes	9 (9.6%)	6 (13%)	3 (6.4%)
Locoregional recurrence	31 (33%)	16 (34%)	15 (32%)

^1^ n (%).

**Table 4:** Survival events in the patients’ cohort according to the H-score-based cutoff by NANOG expression.

Characteristic	Overall, N = 94 ^1^	≤15, N = 48 ^1^	>15, N = 46 ^1^
Death of any cause			
Alive or censored	27 (29%)	11 (23%)	16 (35%)
Dead	67 (71%)	37 (77%)	30 (65%)
Cause of death			
Alive or censored	27 (29%)	11 (23%)	16 (35%)
Death from oral cancer	57 (61%)	30 (63%)	27 (59%)
Death from other causes	10 (11%)	7 (15%)	3 (6.5%)
prDFS			
Censored	18 (19%)	9 (19%)	9 (20%)
Death from oral cancer	36 (38%)	18 (38%)	18 (39%)
Death from other causes	9 (9.6%)	7 (15%)	2 (4.3%)
Locoregional recurrence	31 (33%)	14 (29%)	17 (37%)

^1^ n (%).

**Table 5:** Hazards ratios for different prognostic factors in recurrence specimens using the H-score-based cutoffs of SOX2.

	**prDFS**			**prOS**		
**Characteristic**	**HR** ^1^	**95% CI** ^1^	***p*-Value**	**HR** ^1^	**95% CI** ^1^	***p*-Value**
Age at recurrence diagnosis	1.01	1.00, 1.02	0.2	1.01	1.00, 1.02	0.3
CCI score						
0	—	—		—	—	
1≤	1.36	0.80, 2.32	0.3	1.14	0.64, 2.01	0.7
rT						
rT1	—	—		—	—	
rT2	3.51	1.53, 8.07	**0.003**	3.56	1.36, 9.31	**0.010**
rT3	3.01	1.22, 7.46	**0.017**	5.70	2.09, 15.5	**<0.001**
rT4	2.48	1.22, 5.04	**0.012**	3.58	1.59, 8.05	**0.002**
rTx	2.23	0.97, 5.12	0.059	2.45	0.98, 6.15	0.056
rN						
rN0	—	—		—	—	
rN+/x	1.95	1.11, 3.43	**0.021**	2.87	1.52, 5.39	**0.001**
SOX2_recurrence_H-score						
≤14	—	—		—	—	
>14	0.60	0.35, 1.01	**0.054**	0.54	0.31, 0.94	**0.030**

^1^ HR = Hazard Ratio, CI = Confidence Interval. Bold values indicate statistical significance.

**Table 6:** Hazards ratios for different prognostic factors in recurrence specimens using the H-score-based cutoffs of NANOG.

	**prDFS**			**prOS**		
**Characteristic**	**HR** ^1^	**95% CI** ^1^	***p*-Value**	**HR** ^1^	**95% CI** ^1^	***p*-Value**
Age at recurrence diagnosis	1.01	1.00, 1.02	0.14	1.01	0.99, 1.02	0.4
CCI score						
0	—	—		—	—	
1 ≤	1.23	0.73, 2.09	0.4	1.00	0.57, 1.75	>0.9
rT						
rT1	—	—		—	—	
rT2	4.77	2.07, 11.0	**<0.001**	4.91	1.86, 13.0	**0.001**
rT3	3.19	1.25, 8.17	**0.015**	5.93	2.08, 16.9	**<0.001**
rT4	3.08	1.52, 6.25	**0.002**	4.39	1.91, 10.1	**<0.001**
rTx	2.49	1.09, 5.69	**0.031**	2.79	1.11, 6.98	**0.029**
rN						
rN0	—	—		—	—	
rN+/x	1.92	1.08, 3.43	**0.027**	2.85	1.48, 5.46	**0.002**
NANOG_recurrence_H-score						
≤15	—	—		—	—	
>15	1.11	0.67, 1.85	0.7	1.06	0.61, 1.86	0.8

^1^ HR = Hazard Ratio, CI = Confidence Interval. Bold values indicate statistical significance.

**Table 7:** SOX2 expression in primary and recurrence tumors divided by therapy modality.

**SOX2**			
	**Primary, N = 94** **^1^**	**Recurrence, N = 94** **^1^**	***p*-Value** **^2^**
Median (IQR)	26.631 (1.832, 54.559)	13.963 (2.270, 50.861)	0.621
Missing	26	1	
**Adjuvant RT**			
	**Primary, N = 15** **^1^**	**Recurrence, N = 15** **^1^**	***p*-Value** **^2^**
Median (IQR)	35.061 (8.521, 40.421)	52.670 (12.753, 64.455)	0.28
Missing	2	1	
**Adjuvant RCT**			
	**Primary, N = 21** **^1^**	**Recurrence, N = 21** **^1^**	***p*-Value** **^2^**
Median (IQR)	2.117 (0.394, 26.814)	6.871 (0.738, 14.510)	0.583
Missing	7	0	
**Surgery without Adjuvant**			
	**Primary, N = 58** **^1^**	**Recurrence, N = 58** **^1^**	***p*-Value** **^2^**
Median (IQR)	32.745 (5.406, 63.892)	16.262 (2.441, 45.815)	0.19
Missing	17	0	

^1^ Median (IQR); ^2^ Wilcoxon rank sum test, RT: Radiotherapy, RCT: Radiochemotherapy.

**Table 8:** NANOG expression in primary and recurrence tumors divided by therapy modality.

**NANOG**			
	**Primary, N = 94** **^1^**	**Recurrence, N = 94** **^1^**	***p*-Value** **^2^**
Median (IQR)	42.221 (8.585, 57.148)	8.652 (1.177, 23.368)	<0.001
Missing	27	0	
**Adjuvant RT**			
	**Primary, N = 15** **^1^**	**Recurrence, N = 15** **^1^**	***p*-Value** **^2^**
Median (IQR)	42.663 (22.019, 46.080)	12.123 (1.431, 51.833)	0.555
Missing	2	0	
**Adjuvant RCT**			
Median (IQR)	**Primary, N = 21** **^1^**	**Recurrence, N = 21** **^1^**	***p*-Value** **^2^**
Missing	33.654 (1.852, 43.796)	11.479 (3.231, 18.331)	0.414
	7	0	
**Surgery without Adjuvant**			
Median (IQR)	**Primary, N = 58** **^1^**	**Recurrence, N = 58** **^1^**	***p*-Value** **^2^**
Missing	44.814 (15.930, 61.558)	7.187 (0.943, 23.241)	<0.001
	18	0	

^1^ Median (IQR); ^2^ Wilcoxon rank sum test, RT: Radiotherapy, RCT: Radiochemotherapy.

## Data Availability

The data supporting the findings of this study are not publicly available due to privacy and ethical restrictions.
